# Regenerative Medicine for the Treatment of Ischemic Heart Disease; Status and Future Perspectives

**DOI:** 10.3389/fcell.2021.704903

**Published:** 2021-09-10

**Authors:** Babak Arjmand, Mina Abedi, Maryam Arabi, Sepideh Alavi-Moghadam, Mostafa Rezaei-Tavirani, Mahdieh Hadavandkhani, Akram Tayanloo-Beik, Ramin Kordi, Peyvand Parhizkar Roudsari, Bagher Larijani

**Affiliations:** ^1^Cell Therapy and Regenerative Medicine Research Center, Endocrinology and Metabolism Molecular-Cellular Sciences Institute, Tehran University of Medical Sciences, Tehran, Iran; ^2^Metabolomics and Genomics Research Center, Endocrinology and Metabolism Molecular-Cellular Sciences Institute, Tehran University of Medical Sciences, Tehran, Iran; ^3^Proteomics Research Center, Shahid Beheshti University of Medical Sciences, Tehran, Iran; ^4^Sports Medicine Research Center, Neuroscience Institute, Tehran University of Medical Sciences, Tehran, Iran; ^5^Endocrinology and Metabolism Research Center, Endocrinology and Metabolism Clinical Sciences Institute, Tehran University of Medical Sciences, Tehran, Iran

**Keywords:** heart diseases, ischemia, regenerative medicine, stem cells, tissue engineering

## Abstract

Cardiovascular disease is now the leading cause of adult death in the world. According to new estimates from the World Health Organization, myocardial infarction (MI) is responsible for four out of every five deaths due to cardiovascular disease. Conventional treatments of MI are taking aspirin and nitroglycerin as intermediate treatments and injecting antithrombotic agents within the first 3 h after MI. Coronary artery bypass grafting and percutaneous coronary intervention are the most common long term treatments. Since none of these interventions will fully regenerate the infarcted myocardium, there is value in pursuing more innovative therapeutic approaches. Regenerative medicine is an innovative interdisciplinary method for rebuilding, replacing, or repairing the missed part of different organs in the body, as similar as possible to the primary structure. In recent years, regenerative medicine has been widely utilized as a treatment for ischemic heart disease (one of the most fatal factors around the world) to repair the lost part of the heart by using stem cells. Here, the development of mesenchymal stem cells causes a breakthrough in the treatment of different cardiovascular diseases. They are easily obtainable from different sources, and expanded and enriched easily, with no need for immunosuppressing agents before transplantation, and fewer possibilities of genetic abnormality accompany them through multiple passages. The production of new cardiomyocytes can result from the transplantation of different types of stem cells. Accordingly, due to its remarkable benefits, stem cell therapy has received attention in recent years as it provides a drug-free and surgical treatment for patients and encourages a more safe and feasible cardiac repair. Although different clinical trials have reported on the promising benefits of stem cell therapy, there is still uncertainty about its mechanism of action. It is important to conduct different preclinical and clinical studies to explore the exact mechanism of action of the cells. After reviewing the pathophysiology of MI, this study addresses the role of tissue regeneration using various materials, including different types of stem cells. It proves some appropriate data about the importance of ethical problems, which leads to future perspectives on this scientific method.

## Introduction

Ischemic heart disease (IHD) mostly appears as myocardial infarction (MI) and is one of the most fatal factors for patients around the world (Sahoo and Losordo, 2014; Sharma et al., 2017; Johnson et al., 2019). MI is a pathologic state of the heart that leads to the death of myocytes because of poor blood supply in ischemic conditions (Frangogiannis, 2011). In chronic hypoxic conditions of IHD, some cardiomyocytes are replaced by scar tissue and the others attempt to reduce their energy demands. Moreover, their contractility power also decreases (Fisher et al., 2016). In recent years, regenerative medicine has been widely utilized as a treatment for IHD to repair the lost part of the heart by using stem cells (Fisher et al., 2016). Transplantation of stem cells to the infarcted site can lead to the production of new cardiomyocytes (Michler, 2018). Regenerative medicine is an interdisciplinary approach to restoring, replacing, or repairing the damaged parts of various organs in the body (Glotzbach et al., 2011). Accordingly, different types of stem cells can be used for cardiac cell therapy, including xenogeneic stem cells from nonhuman species, allogeneic ones from human donors, and autologous cells from and implanted into the same person (Alrefai et al., 2015). In other words, it seems that by cell therapy, paracrine factors such as cytokines, chemokines, growth factors, etc. provide an anti-apoptotic and anti-fibrotic state in addition to enhancing endogenous cardiac regeneration and power of contractility (Michler, 2018). Although the therapeutic efficiency of cell therapy makes it a suitable choice for cardiac repair, there is no evidence to show the mechanism of its benefit in the production of heart muscle cells and vessels in humans. It is, therefore, important to explore regenerative medicine and understand the ways it can help in treating cardiac disease. The next step in this field is to find an efficient way of assessing the pros and cons of cardiac cell therapy and recognize if it improves IHD or not. Different categories of strategies are available to evaluate the progression, by using equipment like magnetic resonance imaging (MRI), positron emission tomography (PET), and single-photon emission CT (SPECT) (Dorobantu et al., 2017).

## Basic Pathology of Ischemic Heart Disease

**FIGURE 1:**
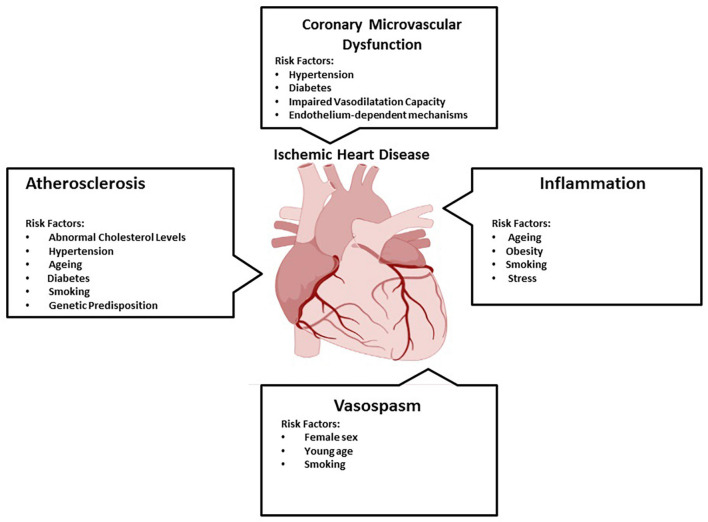
Multifaceted pathophysiology of ischemic heart disease. Ischemic heart disease has multifaceted pathophysiology. Herein, atherosclerosis (the accumulation of fats, cholesterol, etc. in and on the walls of the arteries), coronary microvascular dysfunction (when the small blood vessels in the heart dilate and constrict abnormally), inflammation (an important component of the immune system’s response to infection and injury), and vasospasm (vasoconstriction, or narrowing of the arteries) play a role (Severino et al., 2020).

Ischemic heart disease is one of the first non-communicable diseases in the world and it will become more common in the future. Initial prevention of IHD is not well established. Currently, it accounts for around 45% of all deaths. IHD is a set of syndromes that are closely related and myocardial ischemia is the hallmark of this condition. Atherosclerosis, coronary microvascular dysfunction, inflammation, and vasospasm all play a part in the pathophysiology of IHD (Figure 1; Severino et al., 2020). Because of atherosclerosis, the majority of IHD patients have narrowed epicardial coronary arteries (Buja, 2013; Buja and Vander Heide, 2016). Indeed, IHD is the product of a demand-supply imbalance. In other words, the myocardial tissue does not supply enough blood (Buja and Vander Heide, 2016). Moreover, IHD covers stable and unstable angina, MI, heart failure, and arrhythmia. Herein, typical symptoms are chest pain, cold sweating, nausea, and vomiting (Buja, 2013; Buja and Vander Heide, 2016).

### Inflammatory Response After Ischemia

Endothelial dysfunction, vascular wall inflammation, and lipid accumulation are significant factors in the progression of atherosclerosis (as the most usual causes of myocardial ischemia). The first level of atherosclerosis is the aggregation of LDL and inflammation of the arterial wall. Both the carotid and coronary arteries reach this level. Herein, the expression of vascular cell adhesion molecule-1 (VCAM-1), intercellular adhesion molecule (ICAM)-1, *P*-selection, and *E*-selection along with oxidative stress conditions are induced on endothelial cells during inflammatory responses. The persistence of oxidizing atherosclerotic inducements and pro-inflammatory responses contributes to the activation of T and B cells, more macrophages, and mast cells. Their activation can increase vascular lesions and release cytokines [such as interleukin (IL) 6, IL-1β, interferon (IFN)-α, and tumor necrosis factor (TNF)-α] to enhance monocyte migration into the sub- intimal region (Varbo et al., 2013).

## Enhancing Endogenous Cardiac Regeneration

Recent studies of myocyte turnover have been performed. It is worth noting that the possibility of reconstructing the damaged myocardium after its ischemia is very low. The elements involved in endogenous repairs such as inflammatory cells include macrophages and T cells, cytokines, growth factors, and cardiac progenitor cells (CPCs). Some of the methods that can enhance endogenous cardiac regeneration include modulation of macrophage, regulatory T cell function, induction of myocardial proliferation (it never responds to injuries in adult mammals) (Lauden et al., 2013; Aurora and Olson, 2014; Zacchigna and Giacca, 2014). Additionally, it can be promoted by the administration of fibroblast growth factor-1 (FGF-1), p38 mitogen-activated protein (MAP) kinase, and blocking the Hippo pathway (which includes transcriptional coactivators, serine/threonine kinases, and transcription factors) (Engel et al., 2006; Morikawa et al., 2015).

## The History of Regenerative Medicine Studies in the Field of Ischemic Heart Disease

In recent decades, stem cell therapy has been a very impressive and superior scientific investigational topic. The most promising point in regenerative medicine is that it can present drug-free or surgical-free options for the treatment of patients with chronic pain and severe injuries (Fisher et al., 2016). In the field of IHD, some studies have suggested using stem cells for treatment. Among the different kinds of stem cells, the application of mesenchymal stem cells (MSCs) in the treatment of IHD is more significant and many preclinical and clinical studies have dealt with it (Golpanian et al., 2016). Hereupon, some in vitro findings show that the injection of MSCs to infarcted myocardia can inhibit fibrosis (Zhang et al., 2019). In this respect, bone marrow-derived stromal cell (BMSCs) transplantation along with a combination of bone morphogenetic protein (BMP)-2 and salvianolic acid B (Sal-B) can result in better differentiation of BMSCs to myocardial cells (Lv et al., 2017). In another *ex vivo* study, genetically engineered rat MSCs (modified with Akt) were transferred to ischemic rat myocardium and findings indicated that tissue remodeling was inhibited and most parts of the myocardia were regenerated (Mangi et al., 2003). He et al. (2019) utilized adipose-derived mesenchymal stem cells (ADSCs) for C57BL/6J mice with ischemic myocardia. Additionally, they used resistin-treated ADSCs [ADSC-resistin (adipose tissue-specific secretory factor)] or vehicle-treated ADSCs (ADSC-vehicle) and found that ADSC-resistin had a positive effect on ejection fraction (EF) and reduction of myocyte apoptosis (He et al., 2019). Clinical trials also show that the application of MSCs could be considered a new method in the field of IHD treatment, for instance, in a phase I study by Joshua et al. in which autologous BMSCs were injected into patients with *trans* myocardial revascularization (TMR) (*n* = 10) and coronary artery bypass graft surgery (CABG) (*n* = 4). After 1 year following the patients, regional contractility had been improved in the areas that had cell injection, in comparison to baseline (Chan et al., 2020). In another study, MSCs were injected to patients with chronic myocardial ischemia, left ventricular ejection fractions (LVEFs) of ≤35%, and those who had reversible perfusion defects and were not candidates for revascularization. After following up with the patients for 2 years, significant improvements were detected from baseline to month 12 in several parameters including LVEF, LV end-systolic volume, 6-min walk test and, NYHA functional class (Guijarro et al., 2016). Moreover, an investigation on cell-based therapies for IHD BMSCs led to a reduction in the death of participants who were followed for at least 12 months. In conclusion, as opposed to those who did not receive stem cells, those who received stem cell-based treatments had fewer heart attacks and arrhythmias (Varbo et al., 2013).

## Patient Selection (Comorbidities and Co-Selection)

When researchers design clinical trials about the effects of regenerative medicine in IHD, they have to pay attention to ethical and safety issues such as patient selection (Morikawa et al., 2015). Cautious attention must be given to patient-specific cardiovascular risk factors including age, gender, diabetes, hypertension, smoking, dyslipidemia, depression, psychological problems, and medical help they need (Beckerman et al., 2003; De Groot et al., 2003; Madonna et al., 2016). Studies indicate there is less information about comorbidities and cell therapy in this field (Madonna et al., 2016).

## Controls, Data Reproducibility, Standardization Issue, and Data Quality

The definition of tissue-engineered constructs and combination of cells and biomaterials is considered as an advanced therapy medicinal product definition by European Medicines Agency (EMA) classification (EC No. 1394/2007). There is another definition by the Food and Drug Administration in the United States that covers Human Cells, Tissues, or Cellular and Tissue-Based Products (HCT/Ps). In both of these definitions and to guarantee standardization, safety, traceability, and potency of the final product, it is important to provide criteria according to Good Manufacturing Practice (GMP) production under a manufacturing authorization. Due to the diverse nature of advanced therapy medicinal products, including the tissue-engineered products (TEP), the European Commission (EU) has provided guidelines (e.g., Guidelines on Safety and Efficacy Follow-up: Risk Management of Advanced Therapy Medicinal Products - EMEA/149995/2008) on how to (1) ensure the quality of the production process, (2) evaluate potential risks and, and (3) demonstrate potency and efficacy of the final product via *in vitro/in vivo* tests (Madonna et al., 2019). In the case of cell/material combinations, specific testing of biodegradation and mechanical factors should be done based on the guidelines available, meaning an evaluation of long-term patient/graft interactions needs to be performed (Madonna et al., 2019). For example, there are some recommendations about setting myocardial therapy by using cellular patches. These products should be able to release therapeutic cells without there being inflammatory responses secondary to material degradation. This issue requires patches to be made of fully bio-absorbable materials. These patches have to be resistant to biodegradation (Madonna et al., 2019). Finally, a responsible regulatory authority should observe these products to ensure that the required manufacturing and preclinical strategies have been performed (Madonna et al., 2019).

## Different Types of Mesenchymal Stem Cells Used in this Field

The selection of an appropriate population of cells due to the aim of producing different components of a specific natural tissue – tissue matrix, connective tissue, native tissues- depends on the target structure (Alrefai et al., 2015). Different types of cells have been used in tissue engineering, from stem cells (especially adult stem cells) to terminally differentiated cells but there have been more favorable responses toward using stem cells (Ott et al., 2008; Alrefai et al., 2015) because they can be harnessed more easily than other common cell types (Alrefai et al., 2015; Paschos et al., 2015). In preclinical studies including in vitro and animal experiments, there has been a willingness toward using stem cells. Among all stem cell types, mesenchymal stem cells are one of the most commonly used cell types in this field. Considering preclinical studies, MSC injection in necrotic tissue in acute/chronic MI conditions leads to increased cell proliferation, cell protection against apoptosis and induces angiogenesis in the affected area (Abd Emami et al., 2018; Lu et al., 2019; Sadraddin et al., 2019; Tu et al., 2019; Zhang et al., 2019; Lin et al., 2020). Some studies have applied MSCs in combination with other components such as Nestin and Asprosin, etc. (Lu et al., 2019; Zhang et al., 2019). In some cases, MSCs are used not only to help regeneration in the necrotic area but also to prevent ventricular arrhythmia post-MI (Sadraddin et al., 2019). Studies have shown that applying Insulin-like growth factor 1 (IGF-1) overexpressing MSCs can cause higher rates of cell proliferation and increase cell survival by protecting cells from apoptosis through lowering β-catenin expression (Lin et al., 2020). Thereby, when Asprosin is used in combination with MSCs, it prevents cell apoptosis by reducing oxidizing agents in the hypoxic area (Zhang et al., 2019). Some studies have emphasized the efficacy of Human CD271+ MSCs in preventing post-MI arrhythmia as well as reducing local inflammation (Sadraddin et al., 2019; Sasse et al., 2019). Some other studies have reported the facilitative role of TGF-β, BMP-2, and sal-B in cardiomyocyte differentiation of MSCs (Lv et al., 2017, 2018). Considering the different types of stem cells that are used in clinical experiments, three main types are more commonly applied: Xenogeneic stem cells from nonhuman species; the allogeneic ones from human donors; and autologous cells from the same individual (Alrefai et al., 2015). Allogenic graft transplantation tends to be more successful than the Xeno type, which is due to the immunologic rejections that cause more controversies over using Xenografts. Regarding this issue, more recent autologous grafts have been the subject of increasing attention especially skeletal myoblasts, ADSCs, resident cardiac stem cells (RCSCs), and bone marrow-derived (BMD) stem cells- such as CD34+ cells-, induced pluripotent stem cells (iPSCs), multipotent adult progenitor cells, endothelial progenitor cells (EPCs) and the MSCs that are the main focus of this issue (Alrefai et al., 2015). When coming to clinical studies, the most common function of MSCs is increasing LVEF. Other outcomes are reducing Left Ventricular End Systolic/Diastolic Volume (LVES/DV), better performance in exercise tests as well as promoting cardiac function post-MI (Bartolucci et al., 2017; Florea et al., 2017; Kastrup et al., 2017; Teerlink et al., 2017; Qayyum et al., 2019a). Hepatocyte Growth Factor (HGF) is one of the involved factors in MSC cardiomyocyte differentiation, and cell migration, etc., and is upregulated in MSC transplantation (Bartolucci et al., 2017). Experimental data on the applications of MSC and preclinical studies from 2017 until now is provided in more detail in Tables 1, 2 below. The main allogeneic and autologous cell types and their characteristics will be discussed in more detail later in this paper.

**TABLE 1:** Selected mesenchymal stem cell based clinical studies for ischemic heart disease.

Study	Disease	LVEF	MSC type	Cell origin	Results	References
Chan, 2020	Post revascularization	≤50%	BM	Autologous	↑regional contractility ↑quality of life ↓angina scores at 1 year post-treatment	Chan et al., 2020
Hare, 2020	Chronic IHD	Reduced	BM	Allogenic	↓Infarct Scar Size ↓Peak Oxygen Consumption ↑Six-minute Walk Test ↑MACE ↑Treatment Emergent Adverse Event ↑LVEF ↓Abnormal ECHO Reading	Florea et al., 2017; Hare, 2020
Qayyum, 2019	Chronic IHD	Preserved	AD	Autologous	NS LVEF NS myocardial mass NS stroke volume NS left ventricle end-diastolic volume NS left ventricle end-systolic volume NS amount of scar tissue	Qayyum et al., 2019b
Qayyum, 2019	Chronic IHD	Preserved	AD	Autologous	NC exercise performance ↓performance in METs ↓angina CCS class	Qayyum et al., 2019a
Kastrup, 2017	IHD	≤ 45%	AD	Allogenic	↔ inflammatory parameters ↑cardiac function ↓LVESV ↑left ventricular ejection fraction ↑exercise capacity	Kastrup et al., 2017
Bartolucci, 2017	Chronic IHD	≤40%	UC	Allogenic	↑expression of hepatocyte growth factor (involved in myogenesis, cell migration, and immunoregulation) ↑LVEF ↑New York Heart Association Functional Class ↑Minnesota Living with Heart Failure Questionnaire score	Bartolucci et al., 2017
Teerlink, 2017	Chronic IHD	<35%	BM	Autologous	↓LVEDV ↓LVESV	Teerlink et al., 2017

*BM: Bone Marrow, AD: Adipose-derived, NS: Not Significant, NR: Not reported, IHD: Ischemic Heart Disease, MET: Metabolic Equivalents of Task, CCS: Canadian Cardiovascular Society, UC: Umbilical Cord, MACE: Major Adverse Cardiac Events, LVESV:left ventricular end systolic volume, LVEDV:left ventricular end diastolic volume.*

**TABLE 2:** Some of the mesenchymal stem cells-based preclinical studies for ischemic heart disease.

Study	Type of study	Disease	MSC type	Cell origin	Animal model	Results	References
Lin, 2020	*In vitro*	AMI	BMSCs overexpressing IGF-1	–	–	↑cell proliferation rate ↑migration capacity ↑stemness ↓apoptosis ↑cell survival ↓β-catenin expression	Lin et al., 2020
Gottipati, 2019	*In vitro*	AMI	GFP+ BMMSC	–	–	≠predisposing BMMSCs to aggregation ≠increasing BMMSC susceptibility to phagocytosis ≠heightened immune response ↑cell retention	Gottipati et al., 2019
Zhang, 2019	Animal + *in vitro*	CMI	ASP pretreated BMMSC	Allogenic	C57BL/6 mice	↑LVEF ↓myocardial fibrosis ↑homing of transplanted MSCs NS MSC proliferation and migration ↓H2O2-induced apoptosis ↑SOD2 enzyme ↓H2O2-induced ROS generation ↓apoptosis	Zhang et al., 2019
Shi, 2019	*In vitro*	AMI	serum deprived BMMSCs+Ulinastatin	–	–	↑cell viability ↓apoptosis ↓caspase-3 activation ↓expression levels of Bcl-2, Bcl-extra large and Bcl-associated X protein	Shi et al., 2019
Lu, 2019	Animal	AMI	Nestin positive MSC	Allogenic	Nestin-GFP transgenic mice	↑survival ↑LVEF ↑endogenous CECs ↑chemokine levels	Lu et al., 2019
Tu, 2019	Animal	AMI	BMMSC transfected with miR-15a/15b inhibitors	Allogenic	Luciferase transgenic FVB/N mice	↑proliferation ↓apoptosis ↑VEGFR-2 expression and survival ↑VEGFR-2/PI3K/AKT signaling pathway	Tu et al., 2019
Sasse, 2019	Animal	AMI	H-BM CD271 + MSC	Xenogeneic	SCID beige mice	↓inflammatory cytokines	Sasse et al., 2019
He, 2019	Animal	AMI	ADMSC + resistin	Allogenic	C57BL/6J mice	↑LVEF ↓fibrosis ↓atrial natriuretic peptide/brain natriuretic peptide ↓Apoptosis ↑angiogenesis ↑Cell proliferation and migration	Shi et al., 2019
Sadraddin, 2019	Animal	VA post-MI	H-BM CD271+MSC	Xenogeneic	Immunocompromised Rag2–/– γc–/– mouse strain	↓VA post MI NS scar reduction	Sadraddin et al., 2019
Ciuffreda, 2018	Animal	CMI	BMMSC+ H-HG	Allogenic	Sprague Dawley rats	↓ventricular remodeling ↑neo-vasculogenesis ↑MSC engraftment ↑cardiac function	Ciuffreda et al., 2018
Chen, 2018	Animal	AMI	S1P-treated ADMSC	allogenic	C57BL/6J mice	↑AT-MSCs migration ↓AT-MSCs apoptosis ↑post-MI cardiac function ↑post-MI cardiac remolding	Chen et al., 2018
Abd Emami, 2018	Animal	AMI	BM, AD	Autologous	New Zealand rabbit	↓scar size ↑LVEF	Abd Emami et al., 2018
Lv, 2018	*In vitro*	AMI	BMMSC+TGF-β+Sal-B	-	-	↑expression of cardiac-specific markers ↑The amount of cardiomyocytes differentiated from MSCs	Lv et al., 2018
Lv, 2017	*In vitro*	AMI	BMMSC+BMP-2+ Sal-B	–	–	↑expression of cardiac-specific markers ↑The amount of cardiomyocytes differentiated from MSCs	Lv et al., 2017
Qin, 2017	Animal	AMI	Transfected BMMSCs with hERL and VEGF165	Allogenic	SD rat	hERL/F-FES could be used as a reporter gene/probe system	Qin et al., 2017

*AD: Adipose Derived, S1P: sphingosine 1-phosphate, AMI: Acute Myocardial Infarction, BM: Bone Marrow, ADMSC: Adipose Derived Mesenchymal Stem Cell, BMMSC: Bone Marrow Mesenchymal Stem Cell, TGF-SS: Transforming Growth Factor beta, Sal-B: salvianolic acid B, IGF-1:insulin-like growth factor-1, BMP-2: Bone morphogenetic protein 2, HG: Hydrogel, CMI: Chronic MI, ASP: Asprosin, SOD-2: Superoxide dismutase 2, GFP: green fluorescent protein, CEC: Circulating Endothelial Cells, VA: Ventricular Arrythemia, SCID: Severe Combined Immunodeficiency, BCL-2: B-cell lymphoma 2, hERL: human estrogen receptor ligand, VEGF: Vascular endothelial growth factor, F-FES: 16a-18F fluoro-17-estradiol.*

### Allogeneic Sources

### Fetal Cardiomyocytes

Some characteristics make this kind of stem cell an unfavorable candidate for regenerative therapy including immunogenicity, malignant potential, ethical questions, and limited availability (Li et al., 1996; Alrefai et al., 2015).

### Embryonic Mesenchymal Stem Cells

Cells of three embryonic germ layers and intact cardiomyocytes can be produced by human (ESCs) derived MSCs (EMSC) differentiation (Xu et al., 2002; Alrefai et al., 2015). However, there are some negative aspects of using EMSCs in cardiology, including teratoma formation in rodent models (Lee et al., 2009; Alrefai et al., 2015), malignant transformation, and the legal issues surrounding their use.

### Human Umbilical Cord Blood-Derived Cells

These kinds of cells contain a large number of non-hematopoietic stem cells with two suitable properties for the treatment of bone marrow illnesses, which are (1) that they contain less class II human leukocyte antigens and (2) that they do not trigger an immune response (Li et al., 1996; Alrefai et al., 2015). Some experiments have shown that intramyocardial injection of human cord blood-derived cells could act positively in reducing the infarction size (Alrefai et al., 2015).

### Autologous Sources

### Adipose Derived Stem Cells

Adipose derived stem cells contain a heterogeneous mixture of MSCs, hematopoietic stem cells, and EPCs. Their favorable characteristics are availability, easy harvesting, and low cost, which make them effective in improving ventricular function in animal models of MI, and neoangiogenesis activity is also hypothesized in them (Valina et al., 2007; Wang et al., 2009; Alrefai et al., 2015).

### Skeletal Myoblasts

These kinds of cells can make myotubules after engraftment and improve cardiac function in the infarcted myocardium (Taylor et al., 1998; Alrefai et al., 2015). Some trials, such as the Myoblast Autologous Grafting in Ischemic Cardiomyopathy (MAGIC) trial, discuss the feasibility of myoblast injection into the epicardium as well as CABG surgery. These trials have shown that this process could be possible, with likely functional benefits (Dib et al., 2005; Menasché et al., 2008; Alrefai et al., 2015). The only problem in using this cell type is its interference with the electrical activity of the heart’s native tissue due to its belonging to skeletal muscle lineage, i.e., the new graft can make some islands of skeletal muscle cells between cardiomyocytes that cause arrhythmia (Alrefai et al., 2015).

### BMD Stem Cells

Bone marrow derived are the kind of adult stem cells that have been tested and given profitable results after their transplantation in cases of ischemic heart failure due to their versatility and ease of collection (Orlic et al., 2001; Alrefai et al., 2015).

### Induced Pluripotent Stem Cells

Some adult cells can be programmed in a way that expresses embryonic genes and changes into pluripotent cells. They can produce specific types of cytokines that make it possible for the iPSCs to differentiate into smooth muscle cells (SMCs), cardiomyocytes, and vascular endothelial cells (ECs) (Alrefai et al., 2015).

### Resident Cardiac Stem Cells

Resident cardiac stem cells have recently been isolated with the ability to differentiate into several cell types like cardiomyocytes or vascular SMCs (VSMCs). This is opposed to the notion that there is a lack of self-renewal in cardiomyocytes (Beltrami et al., 2003; Messina et al., 2004; Alrefai et al., 2015). If the harvesting technique is perfect, an improvement in left ventricular (LV) function can be observed in rodent models of MI (Alrefai et al., 2015). The most well-studied RCSCs are c-kit+/Lin– cells which show all the properties of induced stem cells. Besides, they are capable of restoring the cardiac pattern and function in animal models of MI as well as promising improvements in patients with ischemic cardiomyopathy (Beltrami et al., 2003; Linke et al., 2005; Urbanek et al., 2006; Bearzi et al., 2007; Tang et al., 2010; Bolli et al., 2011; Alrefai et al., 2015).

## The Importance of Extracellular Matrix (ECM) in Regenerating Normal Cardiac Tissue

Extracellular matrix plays an important role in the function and homeostasis of the tissue. Accordingly, any kind of microenvironment provided in different regenerating approaches has to mimic normal cardiac ECM. For example, three-dimensional (3D) hydrogel scaffolds are generated from decellularized cardiac ECM (Yanamandala et al., 2017; Madonna et al., 2019). Bio-mimicking and the bioactive properties of ECM proteins make them capable of being used as a hydrogel-based scaffold in cardiac tissue engineering, such as in collagen (Tiburcy et al., 2017; Madonna et al., 2019), fibrin (Hirt et al., 2014; Madonna et al., 2019), gelatin (Nawroth et al., 2018; Madonna et al., 2019), hyaluronic acid (Camci-Unal et al., 2013; Madonna et al., 2019), and alginate (Shin et al., 2013; Madonna et al., 2019). There are some shortcomings in the use of these materials; one of them is the possibility of activating post modification fibrosis due to the emerging stiffness-sensitivity of myocardial resident stromal cells (Mosqueira et al., 2014; Madonna et al., 2019). In this respect, ‘bio-ink’ materials, such as gelatin methacryloyl (GelMA) hydrogels have been introduced, that have the quality of sufficient levels of degradation to control the viscoelastic properties of the used materials and as a result, avoid changes in myocardium compliance (Yue et al., 2015; Bejleri et al., 2018; Madonna et al., 2019).

## Myocardial Tissue Engineering Methods

**FIGURE 2:**
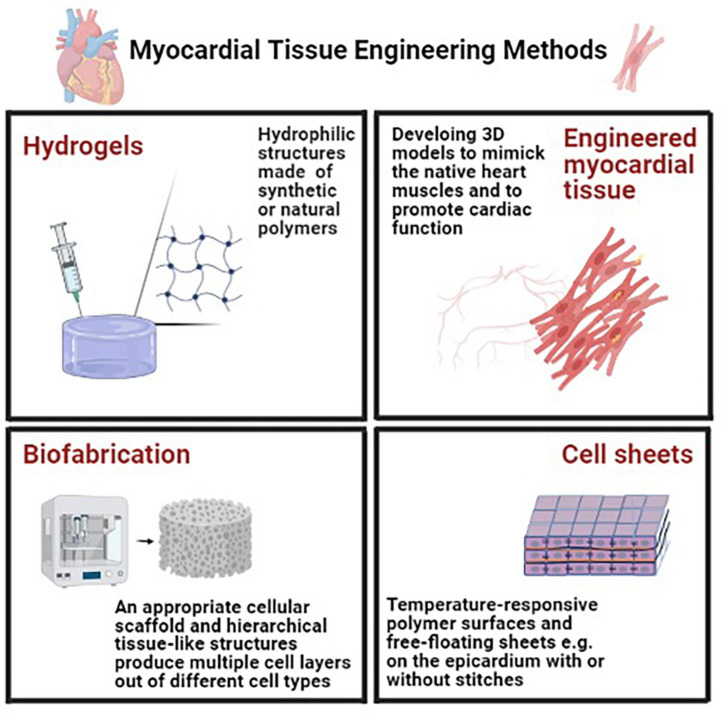
Myocardial tissue engineering methods in order to prolong the graft activity for tissue engineering in ischemic heart diseases several approaches have been arranged including utilizing hydrogels (using hydrophilic structures synthetic or natural polymers), engineered myocardial tissue [developing three-dimensional (3D) *in vitro* models to mimic the native heart muscles], cell sheets (using temperature-responsive polymer surfaces that make the release of cell monolayers possible and can be placed for example on the epicardium with or without stitches) and biofabrication (requiring an appropriate cellular scaffold to provide a cellular microenvironment to serve as a structural platform) (Madonna et al., 2019).

There are several obstacles in the way of tissue engineering in ischemic heart diseases such as low cellular survival, poor localization to the target area. To prolong the graft activity, several approaches have been arranged, including (1) seeding of cells on preformed scaffolds, (2) self-assembly of cells in hydrogels, and (3) cell sheet engineering (Zimmermann, 2009; Weinberger et al., 2017; Madonna et al., 2019). Some of these approaches are discussed in the following section (Figure 2).

### Hydrogels

In this technique, hydrophilic structures, made of synthetic or natural polymers are used (Camci-Unal et al., 2013; Madonna et al., 2019). There are some requirements in this 3D network: the exchange of oxygen, nutrients, and metabolites through their pores, in addition to including growth factors and other molecules to mediate the cross-talk between cells (Wang et al., 2010; Madonna et al., 2019).

### Engineered Myocardial Tissue

There are two main approaches of tissue engineering in cardiology; one of them aims to promote cardiac function and the other is to develop 3D *in vitro* models capable of mimicking the native heart muscles. Some characteristics need to be considered in these approaches: (1) native-like biochemical, electrophysiological, and mechanical cell-ECM and cell-cell interactions, (2) dynamic in vivo like conditions such as fluid flow and shear stress, and (3) correct cell characteristics and morphologies and structural micro-architectures (Madonna et al., 2019).

### Cell Sheets

Temperature-responsive polymer surfaces are used in this approach as they make the release of cell monolayers possible. These free-floating sheets of cohesive cells can be placed on the epicardium with or without stitches. In the same way, 3–4 monolayers can be fused without palpable core necrosis (Madonna et al., 2019). Different types of cells can be used in this approach, such as cardiomyocytes for contractile support and non-myocytes for the delivery of secreted factors (Miyahara et al., 2006; Masumoto et al., 2012; Narita et al., 2013; Madonna et al., 2019). Some shortcomings of this approach include the frailty of sheets that cause folding or tearing, as well as the limited number of sheets that can be stacked on each other without cell death (Madonna et al., 2019).

### Biofabrication

In this approach, to provide a cellular microenvironment to serve as a structural platform, an appropriate cellular scaffold is needed. We follow some purposes by producing such a formation: delivering biochemical factors, providing a suitable environment for cell attachment, migration, and differentiation (Alrefai et al., 2015; Madonna et al., 2019). The main limitation of this approach is that only inhomogeneous cell densities can be achieved because of the cell’s propensity to remain at the scaffold’s surface, so only weakly contracting cardiac tissues can be used in the fabrication method. Accordingly, there has been a lot of effort to make hierarchical tissue-like structures. This specific printing method is a kind of 3D printing (Groll et al., 2016; Cui et al., 2018; Madonna et al., 2019) that produces multiple cell layers out of different cell types (Madonna et al., 2019). There are some standardization issues with this approach, such as correct cellular composition, the positioning of various cell types and materials, vascularization, and the incorporation of bioactive substances (Madonna et al., 2019).

## Delivery Systems and Administration Routes

**FIGURE 3:**
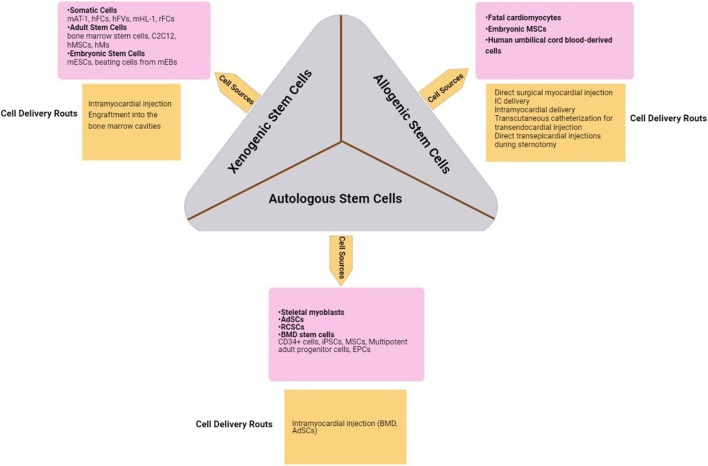
Stem cells are collected from appropriate cell sources. The collected cells are replicated in suitable cultures in order to reduce the risk of tissue rejection. Finally, the generated tissues will be transported to the target organ via different tissue delivery routes due to their availability, invasiveness, and the target tissue’s characteristics (Xiao et al., 2004; Beeres et al., 2007a; Rodrigo et al., 2013; Alrefai et al., 2015; Henry et al., 2017; Konstanty-Kalandyk et al., 2018; Litwinowicz et al., 2018). hFCs, human fetal cardiomyocytes; hFVs, human ventricular cells; hMSCs, human mesenchymal stem cells; hMs, human myoblasts; mEBs, mouse embryoid bodies; mESC, mouse embryonic stem cells; rFCs, rat fetal cardiomyocytes; IC, intracoronary; MSCs, mesenchymal stem cells; AdSCs, adipose-derived stem cells; RCSCs, resident cardiac stem cells; BMD, bone marrow-derived stem cells; iPSCs, induced pluripotent stem cells; EPCs, endothelial progenitor cells.

There are several techniques to deliver stem cells into the myocardium; from direct syringe injection to the left ventricle under visual control to guided percutaneous transendocardial injection to the ischemic area of the left ventricle guided by the NOGA system, which is minimally invasive (Litwinowicz et al., 2018). The direct surgical myocardial injection can be used in hypokinetic myocardial areas, which are not suitable for CABG (Pavo et al., 2014; Litwinowicz et al., 2018). Studies have shown that the largest stem cell retention in the injured myocardium can be provided when this surgery is operated immediately after left anterior descending artery (LAD) occlusion has happened (Elhami et al., 2013; Litwinowicz et al., 2018). Another approach is the intracoronary (IC) delivery of therapeutic agents. Significant drug retention in heart muscle is not provided in this method, but the point is that the extent of stem cell retention in the human myocardium is about 2 h for 1% and 18 h for 5% of the cells, which is very noticeable compared to this extent in the spleen, liver, lung, lymph nodes, and bone marrow (BM) (Litwinowicz et al., 2018). Intramyocardial delivery is a method that has two components: transcutaneous left heart catheterization for transendocardial injection, which is minimally invasive (Tse et al., 2003; Litwinowicz et al., 2018), and direct transepicardial injections under direct visualization during sternotomy (Hamano et al., 2001; Litwinowicz et al., 2018) or left small thoracotomy (Konstanty-Kalandyk et al., 2018; Litwinowicz et al., 2018). Minimally invasive access to the heart muscle is a safe procedure but its main limitation is that it makes safe access to only the anterior and anterolateral wall of the heart possible (Litwinowicz et al., 2018). Full sternotomy has some shortcomings, too. For example, it is associated with some perioperative complications such as bleeding, infections, abnormal sternal healing, or respiratory complications (Litwinowicz et al., 2015, 2016, 2018). Recent animal studies have shown that intracoronary stem cell delivery decreased absolute myocardial blood flow and consequently an increase in myocardial expression of the oxidative stress marker matrix metalloproteinase-2. It reduced the number of CXCR4 receptors and myocardial homing and angiogenic factor release in comparison to intramyocardial cell delivery (Litwinowicz et al., 2018; Zlabinger et al., 2018; Figure 3).

## Stem Cell Homing

Homing is the ability of stem cells to find their destination in the target organ when moving in the bloodstream (Zhao and Zhang, 2016; Tao et al., 2018). Signaling factors direct cells toward their destination, making homing possible (Tao et al., 2018). The new tissue’s phenotype is directed by different signaling factors that can be discovered by observing those involved in native tissue formation. Cell metabolism, migration, and organization will be influenced by these factors (Alrefai et al., 2015). These factors are molecules like chemokines or growth factor receptors on the surface of stem cells to which chemokines, adhesion molecules, growth factors, and the enzymes released from a specific tissue or organ bind (Tao et al., 2018). There are two major types of stem cell homing: endogenous homing and exogenous one after cell transplantation (Tao et al., 2018).

### Endogenous Homing Mechanism

In this mechanism, stem cells mobilize toward their destination while inflammatory factors released after MI direct them. For example, activating proteolytic enzymes [like matrix metalloproteinases (MMPs)] can direct EPCs isolated from bone marrow, toward the infarcted part of the heart (Aicher et al., 2005; Tao et al., 2018). Relevant studies have shown that Hematopoietic stem cells (HSCs) will be mobilized very soon post-acute myocardial infarction (AMI). Consequently, the number of erythroid burst-forming units and granulocyte-macrophage colony-forming units will increase after MI (Wojakowski et al., 2012; Tao et al., 2018). Du et al. (2012) have shown that ESCs homing would be impossible without CD18 and its ICAM-1 (Wu et al., 2006; Tao et al., 2018).

### Exogenous Homing Mechanism

In a specific experiment, MSCs were transduced with human IGF-1 and injected into a rat heart model of AMI. It was concluded that more expression of IGF-1, causes BMSCs to be mobilized through the paracrine activation of stromal cell-derived factor 1 (SDF1)/CXCR4 signaling (Haider et al., 2008; Tao et al., 2018). Another study showed that when vascular endothelial growth factor (VEGF) -expressing MSCs are injected into the infarcted myocardium, endogenous cardiac stem cells (CSCs) will be mobilized through SDF1α/CXCR4 signaling (Tang et al., 2011; Tao et al., 2018).

### Homing-Related Molecules and Signaling Pathways

There are several cytokines involved in the process of stem cell homing. The most important one is the SDF-1/CXCR4 signaling pathway, which plays a crucial role in EPCs, BMSCs, MSCs, and CSCs stem cell populations (Wang et al., 2006; Chavakis et al., 2008; Zhao et al., 2009; Sharma et al., 2010; Herrmann et al., 2015; Sun et al., 2016; Tao et al., 2018). This signaling cascade, phosphatidyl-inositol 3-kinase (PI3K)/Akt, and mitogen-activated protein kinase (MAPK) are involved (Ratajczak et al., 2006; Tao et al., 2018). FMS like tyrosine kinase 3 (Flt-3) ligand, stem cell factor (SCF), IL-6, HGF, IL-3, and as a result CXCR4 protein, are kinds of cytokines for which surface expression increases in the process of MI (Shi et al., 2007; Tao et al., 2018). SDF-1 is another signaling factor that is expressed more than normal conditions, while MI is happening in rats (Pillarisetti and Gupta, 2001; Askari et al., 2003; Tao et al., 2018). The heart’s response to MI is an increase in hypoxia-inducible factor 1 (HIF-1) and VEGF levels, resulting in stem cell mobilization (Mirahmadi et al., 2016; Tao et al., 2018). In an experiment designed by Huang et al. (2014) the increased expression level of VEGF and SDF-1α, angiogenesis, as well as decreased cardiomyocyte apoptosis at the peri-infarct site were reported in post-MI rats’ hearts, transplanted by MSCs (Huang et al., 2014; Tao et al., 2018). Another factor that plays an important role in the migrating and homing process of 10 cardiosphere-derived Lin–c-Kit+ progenitor cells (CLK) is hypoxic preconditioning (HP), which promotes CXCR4 expression through the SDF-1/CXCR4 axis (Tang Y. L. et al., 2009; Tao et al., 2018). HP expression in BMSCs activates focal adhesion kinase (FAK) by Kv2.1 mediation. The previous process is responsible for the migration and homing of BMSCs (Hu et al., 2011; Tao et al., 2018). In an experiment, a CXCR4 antagonist [AMD3100 (AMD)] was used and it was used. Accordingly, an increase in the EPCs population was reported due to the MMP-9 expression downregulation by VEGF when using AMD (Jujo et al., 2010; Tao et al., 2018). Some other cytokines and signaling factors will be discussed in the following. Another factor that increases its serum levels after MI, is clusterin (CST, a stress-responding protein) (Trougakos et al., 2002; Tao et al., 2018). This factor stimulates the migration of cardiac progenitor cells (CPCs). In addition, a study showed that CXCR4 expression will rise in fetal canine heart CPCs, which are transfected by CST. This study was designed on CST-expressing CPCs, which showed that their stimulation by SDF-1 promotes migration through the PI3K/Akt pathway *in vitro* (Li et al., 2010; Tao et al., 2018). Tang J. et al. (2009) showed that wortmannin, which is the inhibitor of PI3K/Akt, can block VEGF-induced CSC migration (Tao et al., 2018). Another study showed that the process of Sca-1+ CSCs migration when injecting peri-infarct myocardium can be promoted by basic fibroblast growth factor (bFGF), through activating the PI3K/Akt pathway (Ling et al., 2018; Tao et al., 2018). There are also some down regulators for the migration process, which are discussed in the following section of this paper. Hyperglycemia suppresses ERK1/2 and p38 MAPK activities and can prevent CSCs from migrating (She et al., 2012; Tao et al., 2018). Hyperhomocysteinemia suppresses NFkB, ERK1/2, and p38MAPK- related to SCF/c-Kit signaling pathway- activities that cause decreased SCF protein expression leading to migration suppression (Wan et al., 2011; Tao et al., 2018). In summary, there are several pathways involved in the stem cell migrating process but the quality of responding to these processes is under question (Tao et al., 2018).

## Quantification of Homing Efficiency

There have been several methods introduced to quantify the homing efficacy such as species mismatch (Jiang et al., 2006; Tao et al., 2018), radioactive labeling (Kraitchman et al., 2005; Li and Hacker, 2017; Tao et al., 2018), fluorescent labeling (Kawada et al., 2004; Tao et al., 2018), and transduction of cells with reporter genes, such as green fluorescent protein gene (Devine et al., 2001; Tao et al., 2018), superparamagnetic iron oxide (SPIO) labeling (Jasmin de Souza et al., 2017; Tao et al., 2018). There are two main methods: (1) quantifying the level of radioactivity in the target organ (2) quantifying the number of cells labeled by fluorescent, which are discussed in detail in the following section (Tao et al., 2018).

### Quantifying the Level of Radioactivity

In this method, imaging techniques are required such as magnetic resonance imaging (MRI), bioluminescence and fluorescence imaging, positron emission tomography (PET), and single-photon emission CT (SPECT) (Meleshina et al., 2015; Tao et al., 2018). MRI has some merits and demerits similar to other modalities. It has a high spatial resolution (Kraitchman et al., 2003; Seeger et al., 2007) and provides beneficial 3D anatomical information, but has lower sensitivity than PET/SPECT (Kraitchman et al., 2005; Tao et al., 2018). PET/SPECT can trace stem cells through radioactive labeling (Bansal et al., 2015; Tao et al., 2018). One of the limitations of this method is the possibility of causing injury to target cells (Brenner et al., 2004; Tao et al., 2018). Labeled gene tracing is possible using both methods (Sheikh et al., 2007; Tao et al., 2018).

### Quantifying the Number of Labeled Cells

One of the methods used in this field is optical imaging in which fluorescent labeling is involved. In this method, the average number of labeled cells in a fixed microscopic field is counted in a short time and highly sensitive way (Tao et al., 2018). A new study has shown that magnetic polymeric nanoparticles (MPNPs) can be used as a labeling agent for MSCs (Supokawej et al., 2015; Tao et al., 2018) since they are not toxic toward cells (Beeres et al., 2007b; Tao et al., 2018).

## Strategies to Improve Cell Homing, Survival, and the Prevention of Tissue Rejection

The efficacy of cell therapy depends on the quality of the graft’s homing, cell recruitment, and coupling in the target tissue. Accordingly, some methods are necessary in order to promote the graft’s homing or cells’ coupling (Seeger et al., 2007).

### Cell Homing and Survival Improvement

Some cytokines are increased in amount during ischemic heart diseases such as SDF-1 or VEGF. These factors mediate cell recruitment. However, the best cell incorporation when infusing stem cells into the coronary arteries is 10% (Seeger et al., 2007). It is documented that low energy shock waves can exacerbate cytokine expression in the target tissue (Seeger et al., 2007). One of the critical issues in investigating the quality of stem cell homing is the time at which the infraction happened. A study (S. Dimmeler, unpublished data) designed on tracing labeled EPCs when infusing them into an infarcted tissue showed that the EPC uptake in an old infarcted tissue is lower than that of an acute infarcted one (Seeger et al., 2007). SDF-1 is a promoting factor that can play a critical role in chemotaxis and cell migration since it stimulates the CXCR4 receptor, which is expressed on EPC and BMC, as well as the ability to retain proangiogenic cells in the target area (Grunewald et al., 2006; Seeger et al., 2007). It was documented that insulin-like growth factor 1 with biotinylated peptide nanofibers into the infarcted heart muscle, can promote homing efficacy since this method changes the target environment (Davis et al., 2006; Seeger et al., 2007).

### Prevention of Tissue Rejection

One of the most challenging issues in cardiac tissue engineering is to prevent foreign-body host immune response. To address this problem, novel biocompatible, immunomodulatory biomaterials have been introduced, as well as smart biomaterials that prevent this issue by doing immunomodulation. The physicochemical properties of these materials have been modified in a way that can promote biomaterial integration and interaction with reparative immune cells such as macrophages and MSC, as well as the controlled delivery of anti-inflammatory small molecules and cytokines (Vishwakarma et al., 2016; Madonna et al., 2019).

## Cell Differentiation Protocols

In order for human EMSCs to differentiate into cardiomyocytes, BMP4 and recombinant human activin A have to be added. This induces cardiac mesoderm to reproduce the main foundations of embryonic development (Laflamme et al., 2007; Burridge Paul et al., 2012; Alrefai et al., 2015; Yue et al., 2015). There is a protocol by Laflamme et al. (2007) and Alrefai et al. (2015) which produces cardiomyocyte populations with over 50% cardiac purity (consisting of nodal cells, ventricular cells, and atrial cells) by adding BMP4 and activin A. In this protocol, first of all, the expression of Wnt ligands is induced and subsequently in order to have a successful cardiac differentiation, this process should be inhibited (Paige et al., 2010; Alrefai et al., 2015). By doing this procedure a biphasic signaling profile will be formed that can be used to evaluate the efficacy of cardiac differentiation. The purity of produced cardiomyocytes based on this protocol is 50% (Alrefai et al., 2015). Yang et al. (2008) and Alrefai et al. (2015) proposed another protocol. In this protocol, small cell groups called “embryoid bodies” are used, mimicking the 3D setting of an evolving embryo. These cells are exposed to bFGF, activin A, BMP4, VEGF and Dickkopf1 (DKK1). Following this protocol, after 4 days, a progenitor population expressing kinase insert domain receptor and platelet-derived growth factor receptor-alpha can be isolated, as well as low levels of ECs (CD31+), fibroblasts [discoidin domain receptor 2 (DDR2)+], and VSMCs (Alrefai et al., 2015). In another approach, to activate Wnt/β-catenin signaling and mesoderm differentiation, the glycogen synthase kinase 3 (GSK3) inhibitor is used which leads to more than 80% of cardiomyocyte purity (Alrefai et al., 2015).

## Cell and Tissue Rejuvenation

As aging has several effects on cells and tissues, decreasing the number and function of both resident and circulating cells can be noted as its comorbidities (Madonna et al., 2016). Therefore, scientists and even the public community have become interested in using regenerative therapies for tissue rejuvenation, especially in the field of cardiac diseases such as acute MI as an irreversible loss of cells (Povsic and Zeiher, 2016). Nowadays, different pharmacological and physiological approaches recommend that epigenetic factors, signaling pathways, and proteins can be utilized to reverse the process of aging (Rando and Chang, 2012; Rando and Wyss-Coray, 2014; Madonna et al., 2016). One of the examples is the pro-viral integration site for Moloney murine leukemia virus-1 (Pim-1) kinase, the over expression of which influences the elongation of telomerase in CPCs (Siddiqi and Sussman, 2013), as well as its anti-aging and anti-apoptotic properties in CSCs and MSCs (Siddiqi and Sussman, 2013; Madonna et al., 2016). In addition, when telomerase and myocardin genes are overexpressed, it is more possible for cells to survive and their proliferation increases (Madonna et al., 2016).

### Cell-Free Approaches

Different studies have shown that the benefits of transplanted stem cells were mostly because of their paracrine effects and cytokines, in comparison with cell differentiation. Thus, releasing factors from transplanted stem cells attracted researchers to focus on them (Jung et al., 2017; Johnson et al., 2019) and try to imitate the beneficial effects of cell therapy through cell-free *in situ* approaches to cardiac regeneration (Madonna et al., 2016; Micheu, 2019). Mediators causing paracrine effects might embrace episomes, growth factors, and non-coding RNAs. Recently, cell-free approaches have been developed, including extracellular vehicles (EVs) secreted from almost all types of cells including cardiomyocytes that contain exosomes and microvesicles (MVs) (Huang et al., 2016; Madonna et al., 2016). Exosomes are small double-bound vesicles that transfer different secreted substances like proteins, lipids, and mRNAs as communicating vehicles between cells in both physiological and pathological conditions (Sahoo and Losordo, 2014; Huang et al., 2016; Jung et al., 2017; Johnson et al., 2019), such as cardiac repair (Sahoo and Losordo, 2014; Madonna et al., 2016).

## How to Assess the Clinical Benefit of Cell Therapy?

Recently, by using stem cell therapy for cardiac disease, it seems necessary to utilize assessments and endpoints that evaluate the impact of this therapy. They are categorized into some groups, including structural, biological, functional, physiological, major adverse cardiac events (MACE), or quality of life.

Structural endpoints contain different items that are (Dorobantu et al., 2017):

• Left ventricular ejection fraction (LVEF) and left ventricular volumes which mostly are LVEF and are measured as the endpoint of stem cell transplantation (Madonna et al., 2016; Dorobantu et al., 2017). Different methods are applied to measure LVEF and LV volume but MRI and recently, 3D-echography are the most accurate (Hung et al., 2007; Dorobantu et al., 2017).
• Myocardial deformation techniques like strain/strain rates, tissue Doppler echocardiography are used and seem to find more sensitive markers than LVEF after acute MI.
• Infarct size is another structural end-point. Several direct and indirect methods are used to quantify the infarct area but contrast enhanced MRI is considered as the gold standard.
• Myocardial viability is measured by PET and SPECT, low-dose dobutamine echocardiography, and MRI. The PET-CT is the most accurate.
• Myocardial perfusion is the last item of structural end-points. MRI, nuclear imaging including SPECT and PET, and contrast echocardiography can quantify myocardial perfusion.

Biological endpoints include biomarkers such as N-terminal pro-brain type natriuretic peptide (NTproBNP) and inflammatory markers like IL-6, CRP (C reactive protein), and TNF-α. Functional capacity is based on patient performance status and exercise tests like the 6-min walk test and treadmill test, etc. (Dorobantu et al., 2017).

## Combination of Tissue Engineering and Cell Therapy

Only cells or cells and biomaterials are widely used as a way of improving cardiac regeneration and recovery (Kim et al., 2018). Several studies show that a mixture of cells and biomaterials improves cell survival in stem cell transplantation in ischemic heart disease (Huang et al., 2016; Micheu, 2019). They are divided into two groups: in vitro tissue engineering and in situ tissue engineering (Christman and Lee, 2006; Hsiao et al., 2013; Ye et al., 2013; Madonna et al., 2019). *In vitro* tissue engineering manufacture scaffolds that contain cells and biomaterials together. Materials are mostly protein-based like collagen, fibrin, and alginate or synthetic polymers such as polyglycolic acid, polyglycerolsebacate, and polyethylene glycol (PEG) (Ye et al., 2013; Huang et al., 2016). In situ tissue engineering includes scaffold-free strategies that make it possible to directly inject a combination of cells and biomaterials into the injured myocardium. Biomaterials of this group commonly are fibrin glue, chitosan, Matrigel, alginate, self-assembling peptides, collagen, and decellularized extracellular matrices (ECMs) (Hsiao et al., 2013; Huang et al., 2016).

## Safety and Ethical Issues

Based on the classification of EMA, the combination of cells and biomaterials are in the group of advanced therapy medicinal products. Therefore there are some guidelines about the certainty of production process quality, evaluation of potential risks, and utilizing *in vitro* and *in vivo* tests to show the final product’s potency and efficacy (Madonna et al., 2019). About ethical issues of clinical trials, researchers should consider that commercial interests do not influence them to start the trial before being ready to start it. They should also note that patients’ expectations may make them interested to be in the group that gets interventions. It is also significant to predict the probable risks and challenges and consider suitable endpoints for the study (Niemansburg et al., 2013; Madonna et al., 2016).

## Conclusion

In recent years the interdisciplinary and novel approaches of regenerative medicine have been utilized in the treatment of IHD, using stem cells to repair the lost part of the heart. There are some recommendations about using cellular patches setting for myocardial therapy. These include using therapeutic cell releasing products whilst avoiding inflammatory responses secondary to material degradation or biodegradation resistant patches. A responsible regulatory authority should be established to ensure that the required manufacturing and preclinical strategies have been carried out and upheld.

Stem cells can be harnessed more easily than other common cell types and are therefore mostly used in tissue engineering. MSCs are one of the most commonly used cell types in this field. In preclinical studies, MSC injection in necrotic tissue in acute/chronic MI conditions leads to enhanced cell proliferation, cell protection against apoptosis and induces angiogenesis in the stimulated area. Moreover, studies have illustrated that applying MSCs with several factors and substances, for instance, Nestin, Asprosin, IGF-1, and the presentation of cell apoptosis, post-MI arrhythmia. Various types of stem cells containing xenogeneic, allogeneic, and autologous stem cells can be used for cardiac cell therapy. Accordingly, allogenic sources include fetal cardiomyocytes, EMSCs, human umbilical cord blood-derived cells, and autologous sources containing ADSCs, skeletal myoblasts, BMD stem cells, iPSCs, and RCSCs. ECM plays an important role in the function and homeostasis of the tissue. The bio-mimicking and bioactive properties of ECM proteins make them capable of being used as a hydrogel-based scaffold in cardiac tissue engineering, such as collagen and fibrin. On the other hand, one of the shortcomings is the possibility of activating post modification fibrosis due to the emerging stiffness-sensitivity of myocardial resident stromal cells. Furthermore, there are several obstacles in the way of tissue engineering in ischemic heart diseases such as low cellular survival and poor localization to the target area.

To prolong the activity of the graft, several approaches have been arranged, including (1) seeding of cells on preformed scaffolds, (2) self-assembly of cells in hydrogels, and (3) cell sheet engineering and (4) bio-fabrication. Moreover, there are several techniques to deliver stem cells into the myocardium; from direct syringe injection to the left ventricle, direct surgical myocardial injection, IC delivery of therapeutic agents. Minimally invasive access to the heart muscle and full sternotomy are suitable approaches but have some limitations, as after stem cell deliverance, the issue of cell homing arises. Signaling factors direct cells toward their destination, making homing possible. These factors are molecules like chemokines on the surface of stem cells to which chemokines, adhesion molecules, growth factors, and enzymes released from a specific tissue or organ bind. There are two major types of stem cell homing: endogenous homing and exogenous one after cell transplantation. After that, we should quantify the homing efficacy such as the level of radioactivity and the number of labeled cells. For human EmSCs to differentiate into cardiomyocytes, BMP4 and recombinant human activin A have to be added. This induces cardiac mesoderm to reproduce the main foundations of embryonic development. There are several protocols relating to this issue such as the Laflamme protocol and Yang protocol.

Another use of regenerative medicine is for tissue rejuvenation, especially in the field of cardiac diseases such as acute MI. Several studies show that a mixture of cells and biomaterials improves cell survival in stem cell transplantation in ischemic heart disease, which is divided into two groups: in vitro tissue engineering and in situ tissue engineering. Finally, it is significant to predict the probable risks and challenges and consider suitable endpoints for the study.

## Future Perspectives

Ischemic heart disease is still one of the major causes of mortality in human society. Cell-based therapy and regenerative medicine represent a new way to be hopeful about the treatment of IHD. It seems necessary to find the best source of stem cells and the most efficient way of importing them to the injured area of the myocardium. Recently, using biomaterials alone as a cardiac therapy has been discussed (Pascual-Gil et al., 2015; Huang et al., 2016) and more studies are needed to clarify the efficacy of this method. Studies show that the need for discoveries of molecular mechanisms that can be utilized in clinical practice dramatically increases because of low cell attachment to the graft. Moreover, researchers are working to increase the effectiveness of regenerative medicine by a combination of cell and gene therapy. Furthermore, cell sheet therapy is another novel approach that can increase the number of transplanted stem cells in the heart tissue. Pharmacological treatments may also affect the molecular pathways related to the increase in the qualification of cell therapy.

## Author Contributions

BA supervised the project from the scientific view of point and advised on study design. MiA, MaA, and SA-M drafted the manuscript. PPR and MH designed the figures and tables. AT-B participated in the study design and provided final approval of the version to publish. MR-T and RK participated in the study design and interpretation. BL supervised the project and participated in critical review. All authors read, provided feedback, and approved the final manuscript.

## Conflict of Interest

The authors declare that the research was conducted in the absence of any commercial or financial relationships that could be construed as a potential conflict of interest.

## Publisher’s Note

All claims expressed in this article are solely those of the authors and do not necessarily represent those of their affiliated organizations, or those of the publisher, the editors and the reviewers. Any product that may be evaluated in this article, or claim that may be made by its manufacturer, is not guaranteed or endorsed by the publisher.

## Abbreviations

3D: three-dimensional
ADSCs: adipose derived stem cells
AMI: acute myocardial infarction
bFGF: basic fibroblast growth factor
BM: bone marrow
BMD: bone marrow derived
BMSC: bone marrow derived stromal cell
BMP4: bone morphogenetic protein 4
CABG: coronary artery bypass graft
CSCs: cardiac stem cells
CST: clusterin
CLK: Lin–c-Kit+
CPCs: cardiac progenitor cells
CRP: C reactive protein
DDR2: discoidin domain receptor 2
DKK1: Dickkopf1
EMA: European Medicines Agency
EU: European Commission
EMSCs: embryonic mesenchymal stem cells
ECs: endothelial cells
ECM: extracellular matrix
EPCs: endothelial progenitor cells
EVs: extracellular vesicles
FAK: focal adhesion kinase
FGF1: fibroblast growth factor 1
Flt-3: Fms Like Tyrosine Kinase 3
GelMA: gelatin methacryloyl
GMP: good manufacturing practice
GSK3: Glycogen Synthase Kinase
HCT/Ps: Human Cells, Tissues, or Cellular and Tissue-Based Products
HGF: hepatocyte growth factor
HIF-1: hypoxia inducible factor 1
HP: hypoxic preconditioning
HSCs: hematopoietic stem cells
MRI: magnetic resonance imaging
IC: intracoronary
ICAM-1: intercellular adhesion molecule 1
MSCs: mesenchymal stem cells
IFN-alpha: interferon-alpha
IHD: ischemic heart disease
iPSCs: induced pluripotent stem cells
MI: myocardial infarction
LAD: left anterior descending artery
LDL: low density lipoprotein
LV: left ventricular
MACE: major adverse cardiac events
MAGIC: myoblast autologous grafting in ischemic cardiomyopathy
MAPK: mitogen activated protein kinase
MMPs: matrix metalloproteinases
MPNPs: magnetic polymeric nanoparticles
MVs: micro vesicles
NTproBNP: N-Terminal Pro-Brain Type Natriuretic Peptide3
PEG: polyethylene glycol
PET: positron emission tomography
IGF-1: insulin-like growth factor 1
HGF: hepatocyte growth factor
TEP: tissue engineered products
TNF: tumor necrosis factor
RCSCs: resident cardiac stem cells
SCF: stem cell factor
SMCs: smooth muscle cells
SPECT: single-photon emission CT
SPIO: superparamagnetic iron oxide
VCAM: vascular cell adhesion protein
VEGF: vascular endothelial growth factor
VSMCs: vascular smooth muscle cells
hFCs: human fetal cardiomyocytes
hFVs: human ventricular cells
rFCs: rat fetal cardiomyocytes
EBs: embryoid bodies
hMSCs: human mesenchymal stem cells
mESCs: mouse embryonic stem cells
LVEF: left ventricular ejection fraction
PI3K: phosphatidyl-inositol 3- kinase
BCL-2: B-cell lymphoma 2
hERL: human estrogen receptor ligand
F-FES: 16 α-18F fluoro-17 β-estradiol.
